# Books and Magazines

**Published:** 1909-04

**Authors:** 


### Books and Magazines.

Parcimony in Nutrition. By Sir James Crichton-Browne, M. D., LL. D., F. R. S. 12mo, cloth. 75 cents, net. Funk & Wagnalls Company, New York.

This is a decidedly interesting little volume, in which the author, who is an eminent British medical authority, very cleverly and keenly attacks the theories of Horace Fletcher and Professor Chittenden, of Yale, and formulates some good, common sense ideas as to what and how much the normal human being should eat. This is a timely and important topic, and a contribution to dietetic literature by so high an authority as Dr. Crichton-Browne is worthy of attention.

It will be remarked that the publishers spell "parsimony" with a "c." By what authority I do not know.—D.

Seven Hundred Surgical Suggestions.—Practical Brevities in Surgical Diagnosis and Treatment. By Walter M. Brickner, B. S., M. D., Assistant Adjunct Surgeon, Mount Sinai Hospital, New York; Editor-in-Chief, *American Journal of Surgery;* Eli Moschcowitz, A. B., M. D., Assistant Physician, Mount Sinai Hospital Dispensary, New York, and Harold M. Hays, M. A., M. D Third series. Duodecimo; 153 pages. New York: Surgery Publishing Co., 92 William Street. Price, semi-de-lux, $1.00; full library de lux, ooze leather, gold edges, $2.25.

This volume is literally "packed full" of useful and valuable information for the general practitioner or surgeon. Written in short, terse epigrammatic paragraphs, it puts its hints up to the eye of the reader in a manner which makes a lasting impression. In its present and enlarged form it is a gem both as to its contents and as an example of the printer's and bookbinder's art.

Any work which would call for three editions in two years, each larger and better than the previous one, is an indication of its usefulness and popularity, and Seven Hundred Surgical Suggestions surpasses them all. The originality of its contents is in keeping with its elaborate and attractive mechanical make-up, and every doctor should have a copy in his library.

---

Appendicitis and Other Diseases of the Vermiform Appendix. By Howard A. Kelley, M. D. With 215 original illustrations, some in colors and 3 lithographic plates. Philadelphia and London: J. B. Lippincott Co., 1909. Price, $6.00, net; 500 pages.

The first edition of Dr. Kelley's great work was issued by another publishing house in 1905, and had a record sale. Its popularity was immense. The Lippincott Co. now present a new, revised and greatly improved edition, which is as near perfection as art can make it,—in point of text, clear, strong type on the highest grade of book paper and bound substantially, tastefully, artistically. The work is too well known already to warrant a detailed review of its contents. It is an encyclopedia within itself; an exhaustive treatise on the subject, covering every possible phase; history, from the first reported case; anatomy of the appendix, copiously illustrated with most excellent drawings from life, from that of the human embryo of six weeks; physiology, bacteriology, pathology, etiology, clinical history, diagnosis, differential diagnosis, leucocytes in; appendicitis in typhoid, in youth and old age; typhlitis, treatment previous to operation; operative treatment, abscess in the vicinity of; peritonitis, care of patient after operation, and postoperative sequelæ; appendicitis in gynecology and obstetrics; neoplasms; specific infections of appendix; hernia of; medico-legal aspects of. Under each of these twenty-five heads the lecture is clear and full and leaves absolutely nothing to be said on the subject. D.

